# Supplementary material for: Antimicrobial resistance and molecular detection of extended-spectrum ß-lactamase (ESBL)-producing Escherichia coli in municipal wastewater in Marrakech
Source: PLoS One. 2025 Jun 16;20(6):e0325816. doi: 10.1371/journal.pone.0325816 (PMC12169590; doi:10.1371/journal.pone.0325816)
Supplement: Attachment — Submitted filename: Response to Reviewers .docx [file pone.0325816.s002.docx]

**Response Letter**

**Manuscript ID:** [PONE-D-24-59416] - [EMID:369bcfd55c8315d9]

Dear Editor,

We would like to thank you for giving us the opportunity to revise our manuscript titled "Antimicrobial Resistance and Molecular Detection of Extended-Spectrum β-Lactamase (ESBL)-Producing *Escherichia coli* in Municipal Wastewater in Marrakech". We would like to thank the reviewers for their careful reading and thoughtful comments. We have carefully taken their comments, corrections, and suggestions into consideration in preparing our revision, which has resulted in a paper that is clearer and more compelling. All modifications are highlighted in the attached manuscript (text highlighted in yellow color).

Sincerely yours,

1.We note that your Data Availability Statement is currently as follows: [All relevant data are within the manuscript and its Supporting Information files.] Please confirm at this time whether or not your submission contains all raw data required to replicate the results of your study. Authors must share the “minimal data set” for their submission. PLOS defines the minimal data set to consist of the data required to replicate all study findings reported in the article, as well as related metadata and methods (<https://journals.plos.org/plosone/s/data-availability#loc-minimal-data-set-definition>).

**Response 1:** All relevant data are within the manuscript. We confirm that our submission contains all raw data required to replicate the results of our study, including the minimal data set .

2. If there are ethical or legal restrictions on sharing a de-identified data set, please explain them in detail (e.g., data contain potentially sensitive information, data are owned by a third-party organization, etc.) and who has imposed them (e.g., an ethics committee). Please also provide contact information for a data access committee, ethics committee, or other institutional body to which data requests may be sent. If data are owned by a third party, please indicate how others may request data access.

**Response 2 :** We confirm that there are no ethical restrictions on sharing the data set used in this study, as no personal or sensitive information is included. Our study did not involve human subjects, and no ethics approval was required.

**Reviewer(s)' Comments to Author :**

**Reviewer 1**

The title should read “Antimicrobial Resistance and Molecular Detection of Extended-Spectrum β-Lactamase (ESBL)-Producing *Escherichia coli* in Municipal Wastewater in Marrakech: Implications for Public Health and Environmental Contamination.”

**Response 1:** We appreciate your valuable insight; however, we believe the original title maintains a concise and impactful representation of the study's main findings and objectives, which we feel resonates more effectively with the intended audience.

The abstract should be improved by incorporating the following: Revise sentences to avoid redundancy and enhance readability. For instance, the phrase: “emphasizing the persistence of these resistant genes even after disinfection processes” could be streamlined to: “highlighting the limited efficacy of disinfection in eliminating resistant genes.”Emphasize practical applications, e.g., how findings can inform wastewater treatment protocols. Avoid excessive numerical data in the abstract; instead, provide a general overview of key findings

**Response 2:** We recognize the suggestions provided. The abstract has been rewritten to enhance readability and conciseness. We have streamlined the language, reduced redundant numerical details, and added a sentence outlining the practical implications for wastewater treatment protocols.

The introduction could be improved as follows: Enhance the clarity of global relevance by briefly discussing similar findings in other regions.The literature review could benefit from integrating more recent studies (2023–2024) to reflect cutting-edge research on wastewater surveillance. State the research gap more explicitly, emphasizing why Morocco’s wastewater systems need targeted investigation.

**Response 3 :** We have added a brief discussion on recent studies (2023–2024) that report similar findings on ESBL-EC persistence in wastewater, highlighting global challenges in antimicrobial resistance (AMR) management. Additionally, we have explicitly addressed the research gap in Morocco.

The methods could be improved as follow : The rationale for selecting 72 samples could be expanded to address whether this number is statistically sufficient to generalize findings. While the description of tools and techniques (e.g., MALDI Biotyper, RT-PCR) is clear, specific details on reagent sources and controls need clarification to enhance reproducibility. Although the study involves wastewater samples, a brief mention of ethical considerations or permits for sample collection would strengthen compliance reporting. Include a section discussing data reliability and replication, especially for RT-PCR and sequencing steps. Clarify the storage or disposal methods for wastewater samples post-analysis

**Response 4:** The selection of 72 samples was based on a balance between feasibility, resource availability, and logistical constraints, ensuring both statistical power and practical execution of the study. Similar studies analyzing antibiotic resistance genes in wastewater have used comparable or smaller sample sizes while still drawing meaningful conclusions. (please see : doi: 10.1016/j.envint.2022.107171). A new subsection discussing data reliability, replication of PCR and sequencing, as well as the storage/disposal protocol for wastewater samples, has been added.

Tables 1 and Figures 1 and 2 contain excessive detailed data, which might be overwhelming for readers. Results focus heavily on resistance gene profiles but lack an in-depth interpretation of patterns and trends. Simplify tables by consolidating similar categories and highlighting the most relevant data. Include additional visual aids, such as heatmaps, to better illustrate resistance patterns across sample sites. Provide a brief summary for each result to connect data points to the research objectives.

**Response 5:** We sincerely appreciate your insightful suggestions. The tables and figures included in the manuscript present critical findings necessary for interpreting ESBL-producing *E. coli* prevalence, phenotypic characteristics, and resistance patterns. Each figure and table plays a distinct role in illustrating the results, and removing any of them could compromise the depth of the analysis. To maintain focus a summary statement has been added for each result section to connect the data to our study objectives. We have streamlined the formatting of Table 1 and ensured that key resistance gene distributions are summarized.

The discussion could be improved as follows: The section occasionally reiterates results instead of critically analyzing them. Lack of detailed suggestions on how findings could influence wastewater treatment improvements. Does not address the limitations of the study, e.g., the exclusion of seasonal variations or genetic typing limitations. Critically evaluate the persistence of genes post-disinfection and propose possible mechanisms.

Highlight practical interventions for improving wastewater treatment protocols based on findings.Discuss broader implications for global health surveillance, aligning with the One Health approach.Explicitly state study limitations and suggest future research directions, such as assessing resistance dynamics across seasons or expanding to other wastewater sites.

The conclusion could be improved as follows: Add a call to action for policymakers to tighten water treatment regulations. Suggest the adoption of advanced treatment technologies or routine monitoring programs. Highlight the need for interdisciplinary collaborations to address antibiotic resistance in wastewater.

**Response 6:** The discussion has been extensively revised to critically analyze the findings, acknowledge study limitations, and propose potential interventions. The conclusion includes a clear call to action for policymakers, recommendations for treatment technologies, and a discussion on the importance of interdisciplinary approaches to combat antibiotic resistance.

The Figures and tables could be improved as follows: Ensure figures are labeled with legends that clearly explain all components. Add captions that emphasize key takeaways for each table or figure. Reduce repetitive information to maintain reader engagement.The references could be improved as follows: Double-check for citation consistency, particularly with formatting styles. Include a few more references from 2023–2024 to strengthen the manuscript’s currency.

**Response 7:** All figures and tables have been revised with updated legends and captions to enhance clarity. We have also reviewed the reference list and incorporated several recent studies to strengthen the manuscript’s currency.

The manuscript grammar and sentence structures could be improved as follows
Revise long sentences for clarity and avoid excessive technical jargon where simpler language can suffice.Conduct thorough proofreading to eliminate typographical errors.

**Response 8:** The manuscript has been carefully proofread and all identified grammatical and typographical errors have been corrected.

**Reviewer 2**

**Ln 92:** please describe the effluent treatment method. In addition, the Results section mentioned about 2 stages of treatment: biological treatment and UV treatment, so please describe the details in this section.

**Response 1:** In response to your request, we have incorporated a detailed description of the effluent treatment method in the Sampling and Study Locations section to ensure clarity and consistency with the Results section.

**Ln 101 :** Please describe the “E. coli-like appearance” and cite the reference(s).

**Response 2:** Changes made. Please see the revised version. Quote ‘Colonies showing a flat, dry, pink, non-mucoid appearance with a darker pink zone of precipitated bile salts, typical of *E. coli’*.

**Ln 102:** please add the purpose of the MALDI method.

**Response 3:** We sincerely appreciate the reviewer's comment .I referenced established literature that thoroughly describes the purpose and application of the MALDI method. The primary function of MALDI-TOF MS in this study is to accurately identify *Escherichia coli* isolates, which is a well-documented and widely accepted application of this technique. Since the cited references provide detailed explanations of the method's purpose and mechanism, I aimed to maintain a concise narrative to avoid redundancy.

**Ln 105:** antimicrobial susceptibility test was performed on which strains, and on how many strains in total?

**Response 4:** AST was performed on ESBL-producing *Escherichia coli* isolates. We acknowledge the importance of reporting the total number of isolates; however, since the materials and methods section describes the experimental procedures, we believe that the appropriate place to report the final number of ESBL-EC isolates is in the Results section. In the Results section, we explicitly mention the total number of ESBL-producing E. coli isolates (n = 134) identified and subsequently tested for AST.

**Ln 124–129** please add reference(s) for the Double Disk Synergy Test.

**Response 5:** Changes made. We have cited recent studies that describe the principles, methodology, and validation of DDST .

**Ln 131:** please note that “RT-PCR” stands for “Reverse transcription-PCR”, not “Real-time PCR”. Which method did the authors perform? Please also describe the PCR protocol after the boiling lysis step in this paragraph.

**Response 6:** Thank you very much for this well-pointed remarque .We have revised the text to explicitly state “Real-Time PCR” instead of "RT-PCR".We have now included the protocols following the boiling lysis step.

**Ln 134:** why did the authors choose the “susceptible” E. coli strain? How about the resistant strains?

**Response 7:** The susceptible *E. coli* strain was used in this study as a negative control to validate the specificity of qPCR amplification and to ensure that the primers did not produce non-specific amplification

**Ln 139:** what does “positive isolates” mean?

**Response 8:** The term "positive isolates" refers to *E. coli* isolates that tested positive for the presence of beta-lactamase genes (blaCTX-M, blaTEM, or blaSHV). To enhance readability, we revised the text to: Quote ‘Isolates that tested positive for beta-lactamase genes’

**Ln 139–142:** the authors mentioned about DNA sequencing and BlastN and BlastP analysis, but no data of these analyses were reported in the Results section!

**Response 9:** Thank you for your valuable feedback. In our study, we conducted a basic BLAST search to compare the obtained sequences with those in the NCBI and ARG-ANNOT libraries. Our objective was to verify the identity and similarity of the sequences rather than to discover novel sequences or mutations. In all cases, the obtained sequences showed 100% similarity to the corresponding sequences already present in these databases. Given this high level of similarity and the fact that no new or unique sequences were identified, we did not find it necessary to include detailed sequence data or submit the sequences.

**Ln 149:** please add the percentage (%) of resistant E. coli out of all resistant strains.

**Response 10:** We have added the percentage of resistant E. coli among all resistant isolates for clarity. As Total resistant isolates = 364 and Resistant *E. coli* isolates = 264 , This means 72.53% of all resistant isolates were resistant *E. coli*. The paragraph has been restructured for better readability while maintaining the logical flow of data.

**Ln 150–151:** typo mistakes, please add a word space before and after “;”

**Response 11:** Changes made. Thank you**.**

**Ln 155 :** please add the percentage (%) of ESBL-EC out of the resistant EC.

**Response 12:** We have calculated and incorporated the percentage in the text. Quote ‘ The phenotypic detection and the confirmation with selective Chrom ID ESBL resulted in the identification of 134 ESBL-EC (50.76%) among the 264 resistant *E.coli*. ’

**Ln 155–159:** was there any strain that was positive with Chrom ID ESBL but showed negative results with the Double Disk Synergy Test?

**Response 13:** We sincerely appreciate the reviewer's insightful comment .We did not observe any isolates that were positive on CHROMID® ESBL Agar but negative with DDST .

**Ln 168–169:** “Fifty percent of the isolated strains showed resistance to 3rd generation cephalosporins”, since the authors performed several steps with different methods in this study, please clarify the method and the number of “isolated strains” in this context.

**Response 14:** Thank you for your comment. In this context, the "isolated strains" refer specifically to the ESBL-producing E. coli (ESBL-EC) gathered after the phenotypic studies, including initial screening and confirmation using selective Chrom ID ESBL. Out of the 264 resistant E. coli strains, 134 (50.76%) were confirmed as ESBL-EC.

**Ln 171–172:** grammar error: “50% trimethoprim/sulfamethoxazole”. grammar error: “Meropenem at 1.56 % were less frequently observed”

**Response 15:** We have corrected two grammatical errors in the text

**Ln 173:** “78.40% (n = 98)” means that 100% were 125, but no population of 125 strains appeared from the previous results. So, from which population was 78.40% calculated? Meanwhile, the number written in the Abstract was 78.35% (n = 105). Please make consistency.

**Response 16:** The percentage of MDR-resistant bacteria was calculated based on a total population of 134 isolates used specifically for AST , out of which 105 were MDR-resistant. It is important to clarify that n=125 refers to the number of isolates analyzed for molecular analysis, which is a different population from the 134 isolates used for AST. This distinction has been clearly stated in the manuscript to avoid confusion.

**Ln 175:** “was covered”: did you mean “was discovered”?

**Response 17:** We changed the word to ‘was observed’.

**Ln 187 and Table 1:** the number of ESBL-producing E. coli was 134 but data in Table 1 showed n = 125. Please make a consistency.

**Response 18 :** A total of 134 ESBL-producing *E. coli* isolates were identified based on phenotypic confirmation using the Double Disk Synergy Test (DDST). However, only 125 of these isolates underwent molecular analysis. The difference (9 isolates) was due to low amplification efficiency, making them unsuitable for molecular characterization.

**Ln 187,188:** data from Table 1 did not support this statement!

**Response 19:** Changes made. We corrected the right values

**Ln 188-191:** these statements should be carefully revised after revising Table 1

**Response 20:** The text in the Results section has been rewritten to accurately reflect the data in the updated Table 1.

This table should be seriously revised and presented in another format considering the following flaws:

- Please provide the number of isolates from each site (site 1,2,3).
  Suppose that the data of blaCTX-M is correct, considering the equality of the total number of blaCTX-M in the three sites (96 + 17 + 12 = 125) and the total isolates (n =125), the number of isolates from site 2 should not excess 17 and the number of isolates from site 3 should not excess 12. However, in the blaTEM row, the respective numbers from site 2 and site 3 were 31 and 15. Similarly, the rows of blaCTX-M+TEM+SHV or blaCTX-M+TEM also showed excessive numbers of isolates at site 2 and site 3. Please explain the reasons for the inconsistent data.
- The calculation of prevalence (%) was difficult to understand, e.g., the prevalence (%) of blaCTX-M from site 1 was calculated out of the total 125 isolates (96/125 = 76.8%), but the prevalence (%) of blaCTX-M-1 from site 1 was calculated out of 96 blaCTX-M-positive strains from site 1 (35/96 = 36.45). These could further lead to misinterpretation and misleading conclusions. Data from the last 3 rows was difficult to understand and was unlogic, for example, the number of blaCTX-M+TEM+SHV (the 3rd row from the bottom) at site 2 was 40, while the number of CTX-M (the 3rd row from the top) at site 2 was only 17.

**Response 21:**

- Thank you for your feedback. The discrepancy resulted from an error in data categorization, particularly in accounting for isolates carrying multiple resistance genes. We evaluated the dataset, corrected the affected rows, and updated Table 1 to ensure consistent and accurate reporting.
- All the percentages were calculated based on the total number of isolates (n=125) to see the overall distribution of each resistance gene across all sampled sites. This approach was chosen to provide a comprehensive overview of the prevalence of each gene relative to the entire population of ESBL-producing *E. coli* isolates.

**Ln 188–191:** data from Table 1 did not support this statement!

**Response 22:** Thank you for your observation. The statements have been revised to ensure accurate alignment with the data presented in Table 1.

**Ln 11:“**78.35% (n = 105)”: please make consistency with the Results section.

**Response 23:** The abstract has been revised to correctly identify the PCR method used and to ensure consistency in all reported values.

**Ln 8,9:** “RT-PCR” was not a correct name of the method, and results of DNA-sequencing data was not reported in this manuscript.

**Response 24:** The term ‘RT-PCR’ was corrected to ‘PCR’ to accurately reflect the method used. DNA sequencing was included to confirm the identity of the isolates by comparing them with sequences in the NCBI and ARG-ANNOT libraries, which showed 100% similarity.

**Ln 12-19** : The prevalences of different genes were calculated among the ESBL-producing *E. coli* only (n = 125), not among the total isolated strains (n = 364). Additionally, the percentages of blaCTX-M-1 or blaCTX-M-15 were calculated out of the blaCTX-M-positive at each site, while the percentages of blaTEM and blaSHV were calculated out of 125 strains. Therefore, these sentences are arbitrary conclusions and are significantly misleading

**Response 25 :** Thank you for your feedback. The prevalence percentages were recalculated to ensure consistency and accuracy
