# Supplementary material for: Antimicrobial resistance and molecular detection of extended-spectrum ß-lactamase (ESBL)-producing Escherichia coli in municipal wastewater in Marrakech
Source: PLoS One. 2025 Jun 16;20(6):e0325816. doi: 10.1371/journal.pone.0325816 (PMC12169590; doi:10.1371/journal.pone.0325816)
Supplement: Attachment — Submitted filename: Response to Reviewers.docx [file pone.0325816.s003.docx]

**Response Letter**

**Manuscript ID :** PONE-D-24-59416R1

**EMID :** 224006d43d0c6d21

Dear Editor,

We would like to express our sincere gratitude for the opportunity to revise our manuscript entitled *"Antimicrobial Resistance and Molecular Detection of Extended-Spectrum β-Lactamase (ESBL)-Producing Escherichia coli in Municipal Wastewater in Marrakech."* We also extend our thanks to the reviewers for their thoughtful and constructive comments, as well as their valuable suggestions. We have carefully addressed all of the reviewers’ comments in this revised version and believe that the changes have substantially improved the clarity and overall quality of the manuscript. All modifications are clearly marked in the revised document, with the updated text highlighted in yellow for easy reference.

Sincerely yours,

**Reviewer(s)' Comments to Author :**

**Reviewer 4**

**Line 10:** How many samples resulted in 364 resistant *Enterobacteriaceae ?*
**Response 1:** Thank you for your comment. We have clarified this point in the revised manuscript. The updated text now reads : *“From a total of 72 wastewater samples, 364 resistant Enterobacteriaceae isolates were recovered, of which 134 were confirmed as ESBL-producing E. coli.”*

**Line 12 :** “in influent samples” what do you mean by influent samples? These genes are found in the bacteria and are not necessary in the sample. kindly clarify.
**Response 2:** We appreciate your observation. We have revised the sentence to clarify that the resistance genes were detected in bacterial isolates obtained from influent samples. By “influent samples,” we refer to raw wastewater collected at the inlet of the treatment plant, prior to undergoing any treatment processes.

**Line 13**: “Although biological treatment reduced resistant gene abundance.” This statement is not necessarily true as it should refer to the bacteria and not the genes.
**Response 3:** Thank you for your valuable observation. Our reference to a reduction in "resistant gene abundance" was intended to indicate a decrease in the number of bacterial isolates carrying these genes. To improve clarity and accuracy, we have revised the sentence to read: *“Although biological treatment reduced the number of ESBL-producing E. coli isolates harboring resistance genes.”*

**Lines 39-31:** The meaning of that paragraph is not clear. Kindly change the word “However” to “As” to make the statement a little clearer.
**Response 4 :** Thank you for your suggestion. We have revised the sentence to enhance clarity, as recommended. The updated text now reads: *“As wastewater treatment plants (WWTPs) often fail to achieve complete microbial removal, antibiotic-resistant bacteria (ARB) and antibiotic resistance genes (ARGs) may persist in treated effluents.”*
**Line 81 :**“Colonies showing a flat, dry, pink…” From which MacConkey agar plate? The one with or the one without 4 mg/L of ceftriaxone. Kindly make it easy for readers to follow.

**Response 5 :** We sincerely appreciate the reviewer’s comment. We have clarified in the revised manuscript that the described colonies were selected from MacConkey agar plates supplemented with 4 mg/L of ceftriaxone, as these were used specifically to isolate ceftriaxone-resistant *E. coli*.

**Line 101:** “AST results were further confirmed using the Phoenix™ Automated Microbiology System (Becton–Dickinson Diagnostic Systems, Sparks, MD, USA” Why was the AST confirmed again?

**Response 6:** Thank you for your question. In our laboratory, the Phoenix™ Automated Microbiology System is routinely used alongside the disk diffusion method to confirm antimicrobial susceptibility profiles. This dual approach enhances the reliability and reproducibility of results, especially when detecting multidrug-resistant organisms such as ESBL-producing *E. coli*. Therefore, both methods were included in the study to ensure data robustness and quality assurance.

**Line 111-112 :** An interactive result between clavulanic acid and one of the chosen cephalosporins on growth was identified as positive for ESBL production.’ What does that mean; interactive result

**Response 7 :** Thank you for your valuable comment. In this context, “interactive result” refers to the synergistic effect observed during the Double-Disk Synergy Test (DDST). Specifically, a keyhole-shaped enhancement or an increase in the inhibition zone between the clavulanic acid disk and any of the cephalosporin disks (cefotaxime, ceftazidime, or cefepime) was interpreted as a positive result for ESBL production. We have revised the sentence accordingly to improve clarity.

**Line 120 :** check the reference style

**Response 8:** Thank you for pointing this out. The reference style has been corrected according to the journal's guidelines.

**Line 143-146 :** “A total of 72 wastewater samples were collected from influent (n = 25) and treated effluent (n = 47 this section is not clear; how many samples did you take. (25+47=72) . Kindly put into the bracket (), including biologically treated effluent or secondary (n = 24) and tertiary treatment effluent (n = 23).

**Response 9 :** Thank you for your helpful comment. We have revised the sentence for clarity. The updated version now reads: “A total of 72 wastewater samples were collected, including influent (n = 25), biologically treated effluent or secondary treatment (n = 24), and tertiary treatment effluent (n = 23).”

**Line 147- ??**

**Response 10 :** Thank you for your comment. We have clarified this section in the revised manuscript. The 364 resistant *Enterobacteriaceae* isolates were recovered from MacConkey agar supplemented with 4 mg/L ceftriaxone. Among these, 264 isolates were identified as *Escherichia coli* using the MALDI-TOF Biotyper® system and were considered resistant based on their growth on selective media. The revised sentence now reads: *“Out of 364 resistant Enterobacteriaceae isolates recovered from selective agar, 264 were confirmed as resistant E. coli, representing 72.52% of the total resistant isolates.”*

**Line 151:** “This indicates that wastewater treatment, particularly tertiary treatment, effectively reduces the proportion of resistant isolates but does not eliminate them completely.” this sounds like discussion, not result

**Response 11 :** Thank you for your comment. We have revised this sentence to better align with the results section. The updated version now focuses on the observed findings.

**Line 153** :“The phenotypic detection and the confirmation with selective Chrom ID ESBL resulted in the” why was the Chrom ID used after you have ID with MaldiTOf is there a discrepancy how was it solve.

**Response 12 :** Thank you for your insightful comment. There is no methodological discrepancy. MALDI-TOF was employed for species identification of *Escherichia coli*, while CHROMID® ESBL agar was used for phenotypic confirmation of ESBL production among the *E. coli* isolates. This sequential approach is a standard practice, ensuring both accurate species identification and reliable resistance profiling.

**Line 167** :“Fifty percent” This should be numerical .

Your table is a bit unclear, I am struggling to understand prevalence here as opposed to frequency. Do you mean frequency among your samples/isolates or prevalence/There is a clear distinction between these two words, and I will be glad if you can make them clear here.

**Response 13 :** We appreciate your feedback. The values presented in the table reflect the frequency of resistance genes among confirmed ESBL-producing E. coli isolates at each site. To enhance clarity and accuracy, we have replaced the term “prevalence (%)” with “frequency (%)” in the table header and updated the corresponding caption accordingly.

**Line 191-206 :** is more of discussions than results

**Response 14 :** Thank you for your observation. In response, we have revised the text to focus solely on the observed findings, ensuring it is more appropriate for the Results section.

**Line 227 :”** Although chlorine disinfection and UV treatment decrease bacterial loads, research indicates” where was this shown in the result section.

**Response 15 :** Thank you for your comment. We have revised the sentence to specifically refer to resistant isolates, in line with the findings presented in the Results section. The updated version now reads: *“Although chlorine disinfection and UV treatment decrease the number of resistant E. coli isolates…”*
